# Molecular signatures of xenograft colorectal cancer in mice treated with topotecan: A mass spectrometry-based study

**DOI:** 10.1016/j.toxrep.2025.102045

**Published:** 2025-05-14

**Authors:** Yousra A. Hagyousif, Ruba A. Zenati, Nelson C. Soares, Hamza M. Al-Hroub, Farman Matloob Khan, Rizwan Qaisar, Rifat Hamoudi, Raafat El-Awady, Ahmad Y. Abuhelwa, Wafaa Ramadan, Waseem El-Huneidi, Eman Abu-Gharbieh, Karem H. Alzoubi, Yasser Bustanji, Mohammad H. Semreen

**Affiliations:** a Research Institute for Medical and Health Sciences, University of Sharjah, Sharjah, United Arab Emirates; b College of Pharmacy, Department of Medicinal Chemistry, University of Sharjah, United Arab Emirates; c Center for Applied and Translational Genomics (CATG), Mohammed Bin Rashid University Medicine and Health Sciences (MBRU), Dubai Health, Dubai, United Arab Emirates; d College of Medicine, Mohammed Bin Rashid University of Medicine and Health Sciences (MBRU), Dubai Health, P.O. box, Dubai 505055, United Arab Emirates; e Laboratory of Proteomics, Department of Human Genetics, National Institute of Health Doutor Ricardo Jorge (INSA), Av Padre Cruz, Lisbon 1649-016, Portugal; f Comprehensive Health Research Centre (CHRC), NOVA Medical School, University NOVA of Lisbon, Lisbon, Portugal; g Department of Pharmacy Practice and Pharmacotherapeutics, College of Pharmacy, The University of Sharjah, Sharjah 27272, United Arab Emirates; h Department of Basic Medical Sciences, College of Medicine, University of Sharjah, Sharjah 27272, United Arab Emirates; i Department of Clinical Sciences, College of Medicine, University of Sharjah, Sharjah 27272, United Arab Emirates; j Department of Biopharmaceutics and Clinical Pharmacy, School of Pharmacy, The University of Jordan, Amman 11942, Jordan; k College of Medicine, University of Sharjah, Sharjah, United Arab Emirates; l Division of Surgery and Interventional Science, University College London, London, United Kingdom; m Department of Pharmacology and Life Sciences, Faculty of Pharmacy, Universiti Teknologi MARA (UiTM), Puncak Alam Campus, Bandar Puncak Alam, Selangor 42300 Bandar Puncak Alam, Selangor, Malaysia; n Cardiovascular Research Group, Research Institute for Medical and Health Sciences, University of Sharjah, Sharjah 27272, United Arab Emirates; o Space Medicine Research Group, Research Institute for Medical and Health Sciences, University of Sharjah, Sharjah 27272, United Arab Emirates

**Keywords:** Colorectal cancer, Topotecan, Metabolomics, LC-MS/MS-QTOF

## Abstract

**Background:**

Colorectal cancer (CRC) is one of the most common cancers worldwide, yet it continues to have a low survival rate, largely due to the lack of effective treatments. Metabolomics offers new insight into disease diagnosis and biomarkers discovery. The aim of the study is to identify serum biomarkers in a CRC xenograft mouse model treated with topotecan using advanced metabolomics techniques to enhance our understanding and management of the disease.

**Methods:**

The therapeutic potentials of the anticancer drug topotecan on metabolomic alterations in CRC were explored using the UHPLC-ESI-QTOF-MS platform. A comprehensive metabolomic analysis was conducted to compare four different animal groups: HCT-116 CRC xenograft mice treated with topotecan (treated group), vehicle-control HCT-116 xenograft mice (untreated CRC xenograft mice), positive controls (healthy mice injected with topotecan), and negative controls (healthy mice).

**Results:**

The study identified 53 altered metabolites across all four groups (*p-value* < 0.05). Independent T-test revealed that 15 metabolites were statistically significant among vehicle controls and negative controls. Additionally, 20 metabolites showed significant differences between the potential responders to topotecan and the vehicle controls. Moreover, only one metabolite was statistically significant between the positive and negative controls.

**Conclusion:**

The findings provide a detailed characterization of metabolic alterations associated with topotecan treatment in CRC. These insights contribute to a better understanding of the drug’s mechanism of action, which may help predict CRC patients’ response to topotecan and guide the development of personalized therapeutic strategies.

## 1 Introduction

Colorectal cancer (CRC) is one of the most common causes of death among men and women globally (T. [25]). CRC was the fourth-leading cause of cancer death in both men and women younger than 50 years in the late 1990s. Today, it has risen to be the leading cause of cancer death in men and the second-leading cause in women [50]. Despite the rise of multiple screening programs to reduce CRC incidence, nearly 25 % of CRC cases are diagnosed at an advanced stage, with limited therapeutic options [11]. An early diagnosis of CRC is associated with a good prognosis. However, after metastasis, the prognosis becomes poor and the 5-year survival rates are reduced to 10 %, warranting an early diagnosis for improved treatment efficacy [31].

The typical treatments for CRC include surgical resection, chemotherapy, and radiation. For early-stage CRC with localized growth, surgery is the standard treatment to achieve complete removal of the tumor and metastases [37]. A high risk of recurrence after surgery is expected, so patients with stage II CRC with recurrence risk or stage III can be treated with adjuvant chemotherapy using irinotecan and oxaliplatin, in addition to capecitabine or 5-fluorouracil (5-FU) (Van der [26]).

Despite all the advances in cancer management, advanced disease is still associated with poor survival and certain limitations for the standard chemotherapeutics are still occurring [15]. For example, with advanced stages, the total response rate of 5-FU is just 10–15 %. Although the inclusion of other anticancer drugs or 5-FU-based regimens, such as FOLFOX, the response rates increase to 40–50 %, but toxicity also increases[17]. Topotecan, a semisynthetic water-soluble analogue of the parent drug camptothecin, is a non-competitive inhibitor of topoisomerase I (Top I), an essential enzyme for DNA replication. Topotecan exerts its cytotoxic effects during the S-phase of DNA synthesis. In preclinical testing, topotecan was shown to have antitumor activity against a diversity of tumors, such as P 388 leukemia, Lewis lung carcinoma, mouse colon carcinomas 38 and 51, human colorectal adenocarcinoma xenograft HT-29, and colorectal carcinoma xenograft HCT-116 [34]. However, the precise molecular mechanisms governing the therapeutic effects of topotecan in CRC remain partly elusive. Despite advancements, novel therapeutic strategies are urgently needed to improve CRC management. Omics analytical techniques, including genomics, transcriptomics, proteomics, and metabolomics, have significantly advanced early disease diagnosis and the monitoring of treatment outcomes [20]. These techniques enable the identification and understanding of molecular differences that may indicate underlying pathological processes. Specifically, metabolomics provides a snapshot of the metabolic state of a cell or organism, which can reflect changes due to disease, environmental factors, or therapeutic interventions. It can help identify metabolic pathways that are altered in diseases and discover potential biomarkers for diagnosis and treatment monitoring.

We developed CRC xenograft models using immunocompromised animals subcutaneously inoculated with HCT-116 tumor cells, creating a heterotopic model. This model was selected for its simplicity, accessibility, and rapid tumor growth, providing an effective system for identifying novel diagnostic biomarkers for noninvasive CRC diagnosis and predictive biomarkers associated with topotecan treatment. Additionally, our research aimed to elucidate significantly altered metabolic pathways and address the existing knowledge gap in omics concerning the treatment efficacy of topotecan in CRC. By employing the ultra-high performance liquid chromatography electrospray ionization quadrupole time-of-flight mass spectrometry (UHPLC-ESI-QTOF-MS/MS) platform, we obtained high sensitivity and precision in identifying small molecules. This study marks the first effort to characterize serum biomarkers in CRC xenograft mouse model treated with topotecan using this advanced analytical platform, potentially paving the way for breakthroughs in early diagnosis, personalized treatment strategies, and improved clinical outcomes for CRC patients.

## 2 Materials and methods

### 2.1 Reagents and chemicals

HCT-116 cell line was obtained from the Radiobiology and Experimental Radio Oncology Lab, University Cancer Center Hamburg, Hamburg, Germany. Fetal bovine serum, penicillin, and streptomycin were purchased from Sigma Aldrich (St. Louis, MO, USA). Topotecan was obtained from (Sigma Aldrich, St. Louis, MO, USA). Hematoxylin and Eosin were obtained from (Sigma Aldrich, St. Louis, MO, USA). Methanol was purchased from Honeywell (Wunstorfer Strasse, Seelze, Germany). LC-grade formic acid was obtained from Fisher Chemical (Geel, Belgium, UK).

### 2.2 Animal ethical approval and mice groups formation

We used 22 Male (10-week-old) BALB/c nude mice weighing between (26 g - 28 g), maintained in the institutional animal facility of the University of Sharjah. The rights of the animals were conserved throughout the whole study. Animal welfare was monitored through three categories: cage environment, animal behavior, and physical appearance. Food and drink were accessible from the cage floor to encourage eating after each injection. Animal welfare assessments were conducted daily. This study was approved by the Institutional Animal Care and Use Committee (IACUC) at the University of Sharjah (Ethical approval number: ACUC-06–08–2022). The study adhered to the ARRIVE guidelines for the reporting of animal research, and a completed ARRIVE checklist is provided as supplementary material.

The experimental animals were numbered and randomly grouped by number. Then the animals were divided into four groups as follows: CRC xenograft mice injected with topotecan (n = 7), CRC xenograft mice injected with vehicle control (n = 5), healthy mice as a negative control (n = 5), and healthy mice injected with topotecan as the positive control (n = 5). To reduce potential disturbance, laboratory animals were housed in standard animal rooms and fed to SPF grade. In this study, humane endpoints were established to minimize animal suffering. The animals were monitored daily for signs of distress, including severe weight loss, labored breathing, abnormal behavior, and lethargy. If any of these signs were observed, the animal was humanely euthanized in accordance with institutional guidelines.

### 2.3 Preparation of HCT-116 cells for injection and subcutaneous tumor cell line transplantation

Dulbecco’s modified eagle medium (DMEM) media was used to culture the colorectal carcinoma cancer cells HCT-116 as monolayers with 10 % fetal bovine serum and 1 % penicillin/streptomycin (100 μg/mL). All cell cultures were kept at 37 °C in 5 % CO_2_ humidified environments. Every two to three days, the culture media was replaced. Following trypsinization and counting of cell pellets, pellets were mixed with Phosphate Buffered Saline (PBS), and 5 million cells/ mL were prepared. Then, HCT-116 cell injections were prepared so that each mouse would receive 2,500,000 cells via the subcutaneous route [52].

100 μL HCT116 cells (25 × 10^5^ cells) were implanted subcutaneously into the sacral region, and the growth of tumor was monitored. The growth of tumors became visible after 12–14 days. After three weeks, uniform masses of CRC tumor were developed in the mice model subcutaneously and the animals were ready to start the experiment. Animals with tumors were randomly allocated into the two sub-groups, including the topotecan and the vehicle controls.

### 2.4 Preparation of topotecan injections

Topotecan hydrochloride hydrate (C_23_H_26_ClN_3_O_6_. HCl·H_2_O) purchased from Sigma-Aldrich was kindly provided by the Drug Design and Discovery research group. Based on the literature, the administration schedule can significantly affect the pharmacodynamic effects of topotecan (efficacy and toxicity). We performed intraperitoneal injections of topotecan (0.625 mg/kg/day, five days a week, for four weeks), as described previously [19].

Topotecan was dissolved in 2 % DMSO as an organic solvent and 98 % PBS, vortexed for 60 s and stored at −80 °C till the day of the injections.

### 2.5 Mice dissection and collection of blood and tissues

**Fig. 1:**
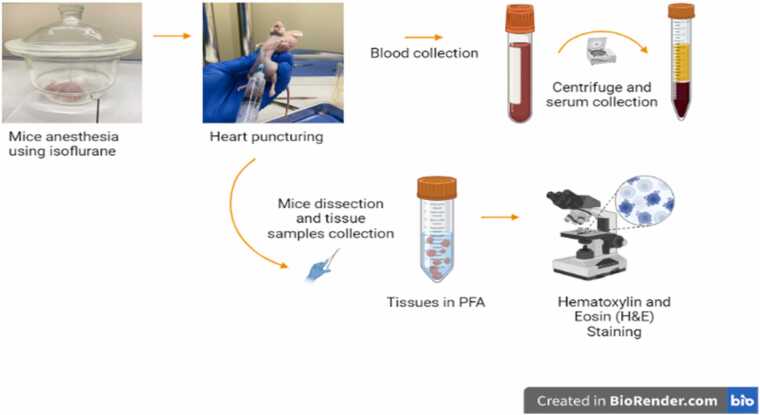
Mice dissection and collection of blood and tissue samples workflow created with BioRender.com.

After 8 weeks from the beginning of the experiment, all mice were sacrificed for blood and tissue collection. First, mice were anaesthetized with inhalational anaesthesia using 3 % isoflurane for induction and 2 % isoflurane for maintenance in a desiccator. Then, by puncturing the heart, blood was immediately collected in an Eppendorf tube, then centrifuged at 14,000 RPM for 15 min to collect serum. Dissection was then performed to collect tissue samples from tumor masses of 12 animals (7 xenografts injected with topotecan and 5 xenografts injected with the vehicle). Tissue samples were collected in an Eppendorf tube containing paraformaldehyde (PFA) to perform Hematoxylin and Eosin (H&E) Staining and compare the presence of cancer cells among different groups, and to conduct tissue metabolomic analysis as future work. Fig. 1 shows the workflow for mice dissection and collection of blood and tissue samples.

### 2.6 Tissue processing and hematoxylin and eosin (H&E) staining

HCT-116 tumor tissues were extracted from mice injected with topotecan, and those injected with vehicle (total of 12 mice) went through several steps during histopathological analyses. Fixation is the first and vital stage in tissue processing [2]. Following tissue removal from the mice, autolysis begins and progresses at varying rates based on various conditions[44]. Using the proper fixative is required to guarantee that the most important histologic characteristics are highlighted. The various fixatives' goal is to stabilize those enzymes and other tissue proteins while also inhibiting microbes, therefore stopping autolysis, with the goal of preserving the tissues as similar to their in vivo state as possible [4].

Hematoxylin and Eosin (H&E) Staining helps in the identification of various cell types and tissues and assists in the diagnosis of disorders such as cancer [36]. Therefore, the histological staining of one representative animal from each experimental group was performed.

### 2.7 Preparation of serum samples for metabolite extraction

**Fig. 2:**
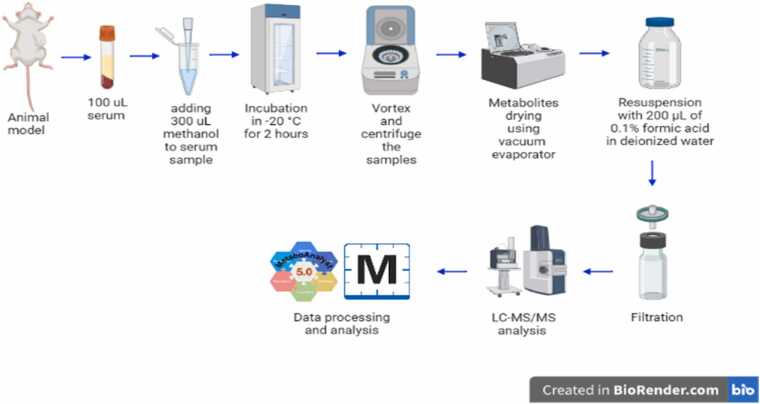
Untargeted metabolomic analysis workflow Created with BioRender.

100 µL of each serum sample was added into the Eppendorf tube, 300 µL of methanol (Wunstorfer Strasse, Seelze, Germany) was added to it, followed by vortex and incubation at −20 °C for 2 h [57]. Next, samples were vortexed and then centrifuged at 14,000 RPM for 15 min. Then, the supernatant was evaporated using speed vacuum evaporation at 35–40 °C. Extract samples were then resuspended with 200 µL of 0.1 % formic acid in Deionized Water-LC-MS CHROMASOLV from Honeywell (Wunstorfer Strasse, Seelze, Germany). Formic acid helps in dissolving the metabolites and donating a proton because the analysis was done in the positive ionization mode. Then, the supernatant was filtered using a 0.45 µm pore size hydrophilic nylon syringe filter for LC-MS/MS analysis and collected in an insert within LC glass vials. Untargeted metabolomic analysis workflow is represented in Fig. 2.

Quality control: An equal amount of each sample (10 µL) was mixed to prepare the quality control (QC) samples. The overlapped base peak ion chromatograms (BPCs) of the QC samples were employed to evaluate the repeatability of the results and the instrument's stability.

### 2.8 Ultra-high performance liquid chromatography coupled to electrospray ionization and quadrupole time of flight mass spectrometry (UHPLC-ESI-QTOF-MS/MS)

A UHPLC (Bruker Daltonic GmbH, Bremen, Germany) coupled to a quadrupole time-of-flight mass spectrometer (QTOF) was utilized to perform untargeted metabolomics analysis. The system was equipped with an electrospray ionization (ESI) source, solvent delivery systems pump (HPG 1300), autosampler, and thermostat column compartment. The mobile phases A (water with 0.1 % formic acid) and B (acetonitrile (ACN) with 0.1 % formic acid) were employed.

A 10 μL aliquot of the sample was injected, and the separation was performed on a Hamilton® IntensitySolo 2 C18 column (100 mm × 2.1 mm × 1.8 μm) at a column oven temperature set at 35 °C. The separation method was approved by Bruker. The first solvent was 99 % water; a gradient elute process was utilized [1]. The more polar metabolites were eluted first, while the less polar ones were retained longer. A microflow approach was employed, utilizing an intermediate flow rate of 0.25 mL/min. For column washing and method conditioning, a flow rate of 0.35 mL/min was used.

The ESI source conditions for every injection were as follows: the drying gas flow rate was

10.0 L/min at a temp of 220 C; the capillary voltage was set at 4500 V; a nebulizer pressure of 2.2 bar. For MS2 acquisition, the collision energy stepping fluctuated between 100 % and 250 %, set at 20 eV and an End Plate offset of 500 V. Sodium formate was used as a calibrant for the external calibration step. The acquisition involved two segments: auto MS scan, which ranges from 0 to 0.3 min for the calibrant sodium formate, and auto MS/MS, which includes fragmentation and ranges from 0.3 to 30 min. The automatic in-run mass scan range was from 20 to 1300 *m/z*, the width of the precursor ion was ± 0.5, the number of precursors was 3, the cycle time was 0.5 s, and the threshold was 400 cts. The method utilized was based on a protocol approved by Bruker since the column and the device were obtained from Bruker.

### 2.9 Data processing and statistical analysis

The data were processed using MetaboScape® 4.0 software (Bruker Daltonics) [39]. Bucketing parameters of the processed data in T-ReX 2D/3D workflow were as follows: intensity threshold of 1000; peak length of 7 spectra; utilizing peak area for quantifying the features. The scan parameters were at a retention time range of 0.3–25 min and a mass range of 50–1000 *m/z*. Each sample was analyzed in duplicate by LC-QTOF (44 injections). Identification of metabolites was based on mapping the MS/MS spectra and retention time in the human metabolite database (HMDB) 4.0 [22], an annotated resource designed to satisfy the needs of the metabolomics community. The compounds with MS/MS were identified using library matching through the annotation process. Then, the selected metabolites were filtered by choosing the set with a higher annotation quality score (AQ score) representing the best retention time values, MS/MS score, *m/z* values, mSigma, and analyte list spectral library.

The metabolites datasets were exported as a CSV file and imported into MetaboAnalyst 5.0 software, a comprehensive platform for metabolomics data analysis [43]. The significance level was set at *q-value* < 0.05, and the fold change was adjusted at 1.5. The principal component analysis (PCA), Sparse Partial Least Squares - Discriminant Analysis (sPLS-DA), volcano plots and enrichment analysis were performed to compare between the four groups. The false discovery rate (FDR) method was utilized to correct multiple hypothesis testing and reduce the rate of false positives.

For statistical analysis, the assumptions of normality and homogeneity of variances were first visually assessed using histograms and boxplots. Histograms were used to check the distribution of data within each group for normality, while boxplots were utilized to evaluate the spread of the data and identify potential violations of the equal variance assumption. Subsequently, one-way analysis of variance (ANOVA) was performed to compare the four groups (xenografts injected with topotecan, xenografts injected with vehicle, negative controls, and positive controls). Independent Student’s t-tests were then conducted for pairwise comparisons between two groups: treated xenografts vs. vehicle xenografts, vehicle xenografts vs. negative controls, and negative controls vs. positive controls.

## 3 Results

### 3.1 Xenograft models characteristics and HCT-116 Tumor growth

All animals included in the study were (10-week-old) males BALB/c nude mice, weighing between (26–28 g). Animals’ behavior and health condition were monitored in a daily basis, and their weights were recorded every other day, throughout the study. Also, tumor masses

weights were recorded at the day of the dissection, see Table S1 shows potential non-responder xenografts with the highest tumor weights in red.

After 3 weeks, uniform masses of CRC tumor were grown in the mice models subcutaneously, compared to initial mass growth after 0, 1 week, and 2 weeks respectively. Then, animals were ready to start the experiment. Xenograft models who were treated with topotecan had a smaller tumor mass than those who received vehicle control, indicating that topotecan treatment was able to shrink tumor growth (see Table S1).

**Fig. 3:**
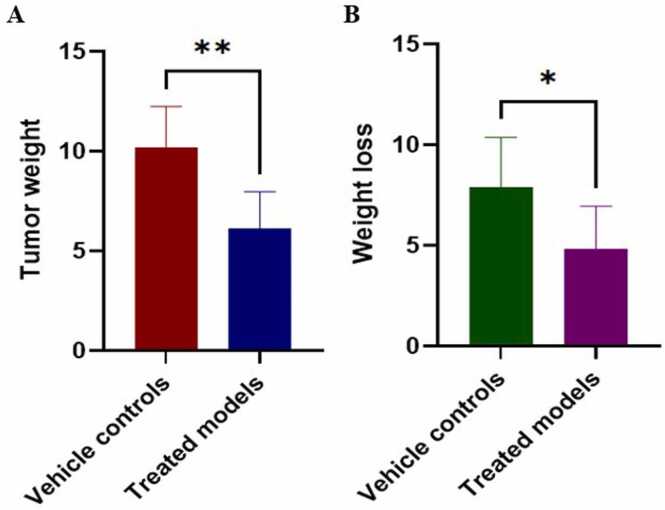
Effect of topotecan treatment on tumor weight and body weight loss in a mouse xenograft model. Tumor weight (A) and body weight loss (B) were measured in vehicle control and topotecan-treated groups. Tumor weight is presented in grams, with a significant reduction observed in the Topotecan-treated group compared to the vehicle control (p < 0.01). Body weight loss, also measured in grams, was significantly lower in the treated group (*p < 0.05*). Data are presented as mean ± standard error of the mean (SEM). Statistical significance was analyzed using an independent Student’s *t*-test, with *p-value* < 0.05 (*) and *p-value* < 0.01 (**).

An unpaired two-tailed t-test was done to compare tumor weight between treated models and vehicle controls. The vehicle controls presented significantly higher tumor weight than treated ones (p = 0.0049) (Fig. 3A). Weight loss between treated models and vehicle controls was also compared, and results showed that vehicle control mice lost significantly more weight compared to treated models (*p* = 0.0435) (Fig. 3B), indicating that intraperitoneal (i.p.) injections of topotecan did not induce a dramatic weight loss, but did shrink tumor growth. In addition, Table S1 shows that positive controls experienced negligible weight loss, which was not exceeding 2.0 g, indicating the safety of the used dose.

### 3.2 Hematoxylin and eosin (H&E) staining

**Fig. 4:**
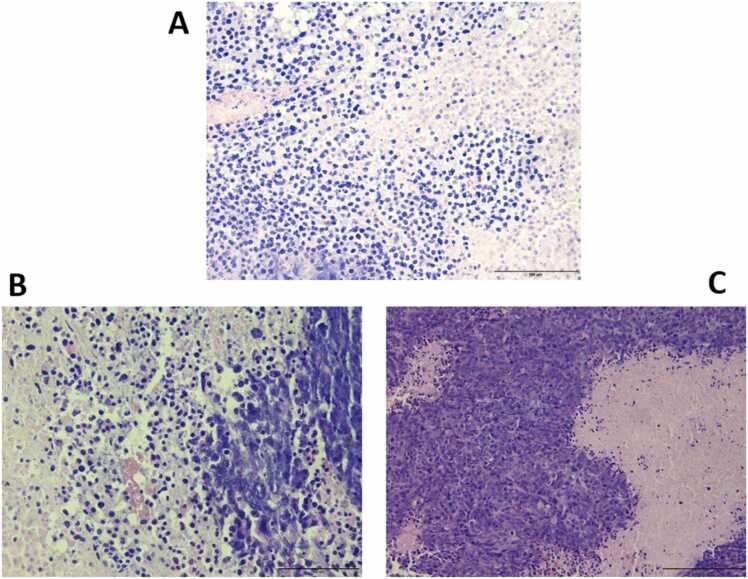
Histological staining of one representative animal from each experimental group. Slides stained with hematoxylin and Eosin (H&E); **(A)** tissue sample from potential responder model; **(B)** tissue sample from potential non-responder model; **(C)** tissue sample from vehicle control model.

Morphologically, a cancer cell's nucleus is frequently abnormal in size and shape. it is often bigger and darker than that of a normal cell under a microscope after being stained with particular dyes, and its size can vary substantially, since it contains an excess of DNA [33]. Fig. 4 shows the histological staining of one representative animal model from each experimental group. It can be clearly seen that tissue sample from potential responder model had the smallest and most uniformly shaped cells under the microscope (Fig. 4 A), followed by the tissue sample from the potential non-responder model, which represents bigger nucleus, irregular size and shape, and darker color. (Fig. 4B). Ultimately, tissue sample from vehicle control model was the most intensely colored with a scarce cytoplasm indicating the high presence of cancer cells (Fig. 4 C).

### 3.3 Metabolic changes

**Fig. 5:**
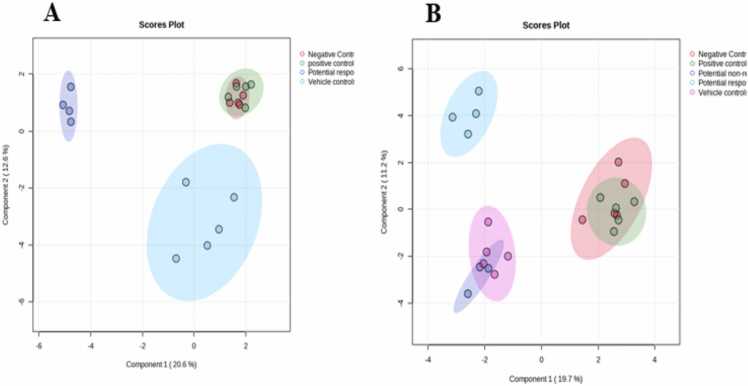
Sparse Partial Least Squares - Discriminant Analysis (sPLS-DA) for **(A)** vehicle controls and negative controls; **(B)** treated xenografts and vehicle controls.

UHPLC-ESI-QTOF-MS detected a total of 152 metabolites. The HMDB was the base for the qualitative identification and filtration of the metabolites. After filtration, a total of 130 metabolites were included in the analysis. Multiple *t*-test analyses with fold change cut-off value = 1.5 were conducted between each pair of groups using MetaboAnalyst software. The direction of comparison was as follows: treated “potential responders” xenografts/vehicle controls, potential responder xenografts/potential non-responder xenografts, and positive controls/negative controls. The sparse partial least squares-discriminant analysis (sPLS-DA) showed separate clusters when comparing potential responder and vehicle controls, representing the big difference between the two groups. Also, full overlapping between negative controls and positive controls indicated the big similarity between the two groups (Fig. 5A). In addition, (sPLS-DA) was performed again between the five groups; potential responders, potential non-responders, vehicle controls, positive controls and negative controls, and showed a partial overlapping between potential non-responders and vehicle controls, indicating some similarity between them. To statistically confirm the similarity between potential non-responders and vehicle controls, a one-way ANOVA test was conducted comparing potential responders, potential non-responders, and vehicle controls. The analysis verified no significant difference between potential non-responders and vehicle controls (Fig. 5B).

The heat map representing the identified metabolites in the 5 groups was also conducted using MetaboAnalyst software. It showed complete separation between potential responders and vehicle controls. Also, vehicle controls and negative controls were well separated. These observations indicate the big difference between the two groups. In addition, an overlapping was observed between positive controls and negative controls. Also, potential non-responders were not well separated from vehicle controls. These observations indicate the similarity between the two groups (Fig. S1). All these observations indicate a substantial change in animals’ metabolic profiles.

#### 3.3.1 Potential biomarkers for CRC diagnosis

**Fig. 6:**
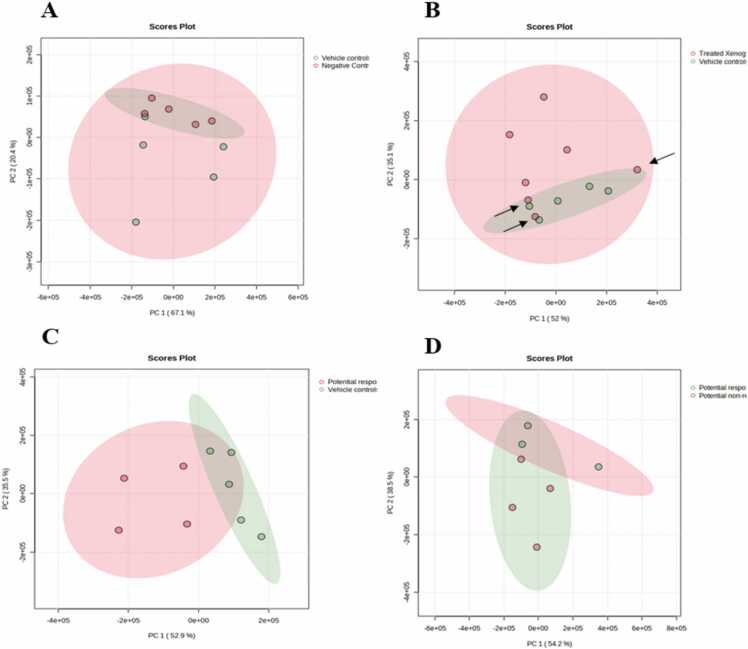
Principal Component Analysis (PCA) for **(A)** vehicle controls and negative controls; **(B)** treated xenografts and vehicle controls; **(C)** potential responders and vehicle controls; **(D)** potential responders and potential non-responders.

**Fig. 7:**
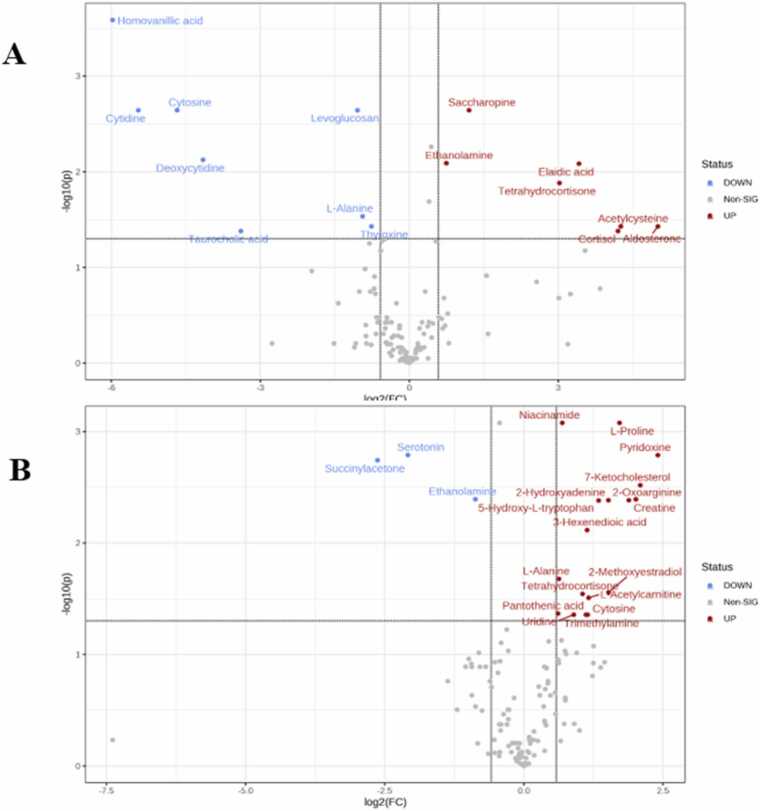
Volcano plots of **(A)** vehicle controls and the negative controls; **(B)** Potential responders and vehicle controls.

First, the *t*-test was done to compare vehicle controls (5 models) and negative controls (5 models). Principal Component Analysis (PCA) for these two groups showed a minimal overlap between the two groups, indicating robust differences between them (Fig. 6A). Fifteen metabolites were found to be statistically significant between these two groups (*q-value* < 0.05) (Table 1). The volcano plot is shown in Fig. 7A.

**Table 1:** The significantly altered metabolites among the vehicle controls and the negative controls.

Metabolite	t.Stat	p value	FDR (False Discovery Rate)	FC (Fold Change)
Aldosterone	4.0594	0.0036365	0.035229	32.1
Acetylcysteine	3.9792	0.0040665	0.035229	19.1
Cortisol	3.7991	0.005243	0.039615	18.4
Elaidic acid	5.536	0.00055	0.007479	10.6
Tetrahydrocortisone	5.0631	0.000973	0.012034	8.1
Saccharopine	7.2934	8.44E−05	0.002296	2.3
Ethanolamine	5.6473	0.000483	0.007298	1.6
Thyroxine	−3.9656	0.004145	0.035229	0.5
L-Alanine	−4.3047	0.002599	0.027192	0.5
Levoglucosan	−7.3633	7.89E−05	0.002296	0.4
Taurocholic acid	−3.8406	0.004943	0.039544	0.09
Deoxycytidine	−5.8346	0.00039	0.007298	0.05
Cytosine	−7.6195	6.19E−05	0.002296	0.03
Cytidine	−8.0427	4.20E−05	0.002296	0.02
Homovanillic acid	−12.167	1.93E−06	0.000262	0.01

#### 3.3.2 Potential biomarkers for treatment and treatment response prediction

Another T-test was done to compare between the 7 treated xenografts (topotecan models) and the 5 untreated xenografts (vehicle controls). It can be clearly noticed in the PCA of these two groups that three treated xenografts are highly overlapping with the vehicle controls, indicating minimal response to topotecan treatment (Fig. 6B). The term potential non-responder has been applied to these three animals, and potential responders has been applied to the other four animals.

Then, these three potential non-responder xenografts were excluded from the analysis, and the *t*-test was repeated between the four potential responders and the five vehicle controls. Among all 130 metabolites, 20 were statistically significant between potential responders and vehicle controls (< 0.05), 17 elevated vs. three reduced (Table 2). The volcano plot is shown in Fig. 7B. PCA in Fig. 6C shows clusters and the absence of overlapping.

**Table 2:** The significantly altered metabolites among the potential responders and vehicle controls.

Metabolite	t.Stat	p.Value	FDR (False Discovery Rate)	FC (Fold Change)
Pyridoxine (vitamin B6)	8.5299	6.04E−05	0.001643	5.3
7-Ketocholesterol	7.3401	0.000157	0.003055	4.26
2-Oxoarginine	6.7517	0.000265	0.004084	4.03
Creatine	6.509	0.000331	0.004165	3.69
L-Proline	10.64	1.42E−05	0.000842	3.29
2-Methoxyestradiol	4.4194	0.003084	0.02485	2.87
2-Hydroxyadenine	6.4914	0.000337	0.004165	2.86
5-Hydroxy-L-tryptophan	6.3929	0.00037	0.004191	2.53
L-Acetylcarnitine	4.2253	0.003911	0.028199	2.24
Cytosine	3.8127	0.006605	0.042379	2.21
3-Hexenedioic acid	5.6961	0.000738	0.007724	2.19
Trimethylamine	3.7841	0.006855	0.042379	2.16
Tetrahydrocortisone	4.3396	0.003398	0.025861	2.07
Uridine	3.8241	0.006507	0.042379	1.86
Niacinamide	10.216	1.86E−05	0.000842	1.61
L-Alanine	4.7114	0.002179	0.019758	1.54
Pantothenic acid	3.9218	0.005736	0.041055	1.53
Ethanolamine	−6.7285	0.00027	0.004084	0.54
Serotonin	−8.8337	4.82E−05	0.001637	0.23
Succinylacetone	−8.1579	8.04E−05	0.001823	0.16

In addition, PCA in Fig. 6D shows the minimal overlapping between potential responders and potential non-responders, and six metabolites were statistically significant between them, four elevated vs. two reduced (Table 3).

**Table 3:** The significantly altered metabolites among the “potential responders” treated xenografts and “potential non-responders” treated xenografts.

Metabolite	t.Stat	p.Value	FDR (False Discovery Rate)	FC (Fold Change)
7-Ketocholesterol	14.74	2.60E−05	0.001178	11.06
2-Oxoarginine	9.959	0.000174	0.005779	8.40
Pyridoxine	8.905	0.000297	0.006788	8.22
L-Proline	9.5561	0.000212	0.005779	3.22
Serotonin	−20.967	4.57E−06	0.000619	0.18
Succinylacetone	−18.239	9.11E−06	0.000619	0.11

#### 3.3.3 Potential biomarkers for safety

**Fig. 8:**
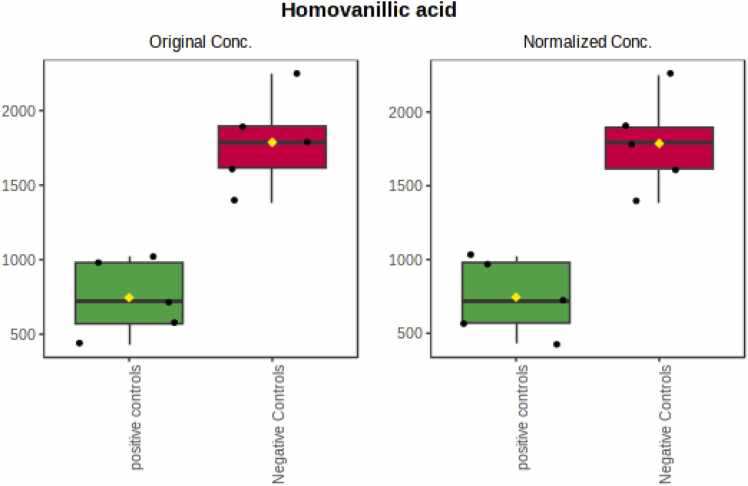
The abundance of Homovanillic acid was statistically significant between the positive controls and negative controls.

T-test performed between positive and negative controls revealed that there is only one significantly reduced metabolite in the positive controls compared to negative ones, which is Homovanillic acid (0.41674-fold), as shown in Fig. 8.

#### 3.3.4 Functional enrichment analysis

**Fig. 9:**
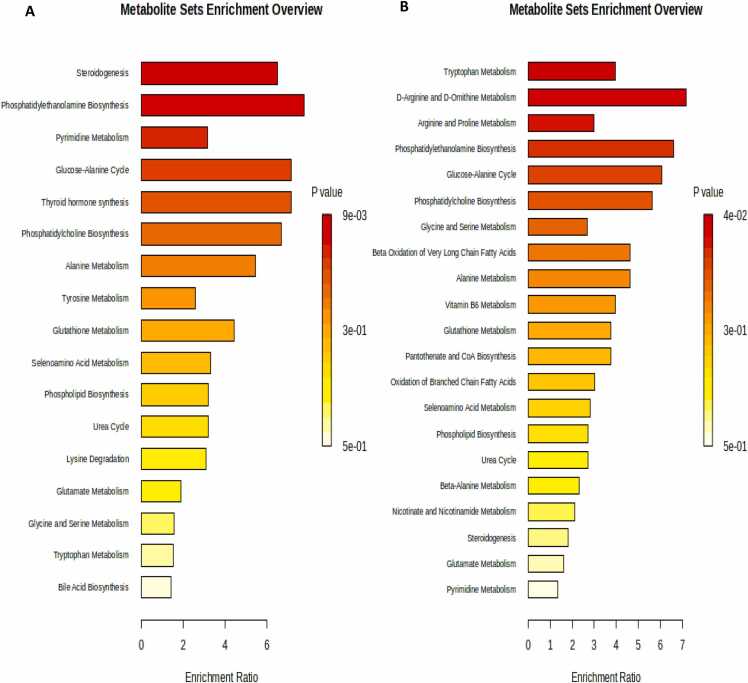
Enrichment analysis of (A) vehicle controls and negative controls (B) potential responders’ groups and vehicle controls.

According to the functional enrichment analysis of the significantly altered metabolites between vehicle controls and negative control, topotecan had a substantial effect on several metabolic pathways such as steroidogenesis and phosphatidylethanolamine biosynthesis (Fig. 9 A). In addition, exploring the therapeutic effect of topotecan between potential responders’ group and vehicle controls revealed that several metabolic pathways were significantly altered, such as tryptophan metabolism (Fig. 9B)

## 4 Discussion

Metabolomics has been used in a variety of research fields, including cancer research and other disorders, due to the rapid progress of mass spectrometry technologies and MS-based omics approaches. It uses novel biomarkers to analyze disease etiology at the molecular scale [27]. This is the first research to examine the altered metabolites and metabolic pathways of HCT-116 xenograft models treated with topotecan. We performed a metabolomic analysis study that compared four groups: HCT-116 xenograft models treated with topotecan, vehicle controls, and positive and negative controls, using an untargeted LC-MS/MS approach. According to the groups overlapping shown in the sparse sPLS-DA (Fig. 5B), treated xenografts were split into two sub-groups, potential responders and potential non-responders, according to their potential response to the treatment. A total of 20 metabolites were significantly associated with potential responders in relation to vehicle controls. These metabolites can be employed as potential biomarkers for CRC management. Also, a total of 15 metabolites were significantly altered among the vehicle controls and negative controls, which can be utilized as potential diagnostic biomarkers for CRC. In addition, six metabolites were statistically significant among the potential responders and potential non-responders, displaying biomarker potential for treatment response prediction. Furthermore, only one metabolite was statistically significant among the positive and negative controls, which can be used in assessing topotecan safety in our setting.

### 4.1 Potential biomarkers for CRC diagnosis

The *t*-test conducted between the vehicle controls (5 models) and negative controls (5 models) identified 15 significantly altered metabolites. Seven metabolites were upregulated, and eight were downregulated in the vehicle controls compared to negative ones. These metabolites demonstrate biomarker potential for the diagnosis of CRC.

Aldosterone is the main mineralocorticoid steroid hormone produced in the adrenal gland. Through binding to the mineralocorticoid receptor (MR) in the connecting tubule and cortical collecting duct in the kidneys, it performs its primary physiological function of preserving sodium and potassium balance and blood pressure regulation [54]. A previous study suggested that MR potentially acts as a tumor suppressor. Also, this study reported that an under-expression of MR is an early event in CRC progression, which is also associated with VEGF receptor overexpression, a pro-angiogenic factor secreted by tumor cells to promote tumor angiogenesis, invasion, growth, and metastasis[12]. Laura Tiberio and colleagues investigated MR expression, patients’ survival, and tumor angiogenesis in patients with CRC. Firstly, they demonstrated that MR expression was decreased and associated with decreased patients’ survival and increased micro-vessel density (MVD). Then, to test the hypothesis that MR stimulation by agonist may negatively control tumor angiogenesis, they used HCT-116 cell line genetically modified to express high levels of MR. After the aldosterone supply, there was a significant decrease in VEGF mRNA expression, which is essential for tumor progression [53]. Our results showed that aldosterone was robustly elevated in the vehicle controls (32.131-fold) compared to negative controls. We hypothesize that the expression of MR was downregulated in the vehicle controls, and elevated aldosterone levels were a response to prevent tumor angiogenesis by activating MR.

The metabolite Acetylcysteine, also known as N-acetylcysteine (NAC), is the acetylated form of the amino acid L-cysteine. The human body can create NAC from other amino acids, particularly L-serine and L-methionine. It is classified as a "semi-essential amino acid." That is, there is no daily NAC demand [18]. NAC has been identified as a promising cancer chemo-preventive agent. It has been recognized in several animal studies to be effective in cancer prevention. Particularly in CRC, there is an overexpression of insulin-like growth factor I receptor (IGF-IR) and epidermal growth factor receptor (EGFR). These receptors contribute to tumor growth and angiogenesis. A previous study investigated whether NAC can affect the expression level of these receptors on several colorectal cancer cell lines (HT29, LoVo, SW480, and KM12SM). Results showed a time- and dose-dependent decrease of EGFR and IGF-IR in CRC cell lines after NAC treatment, leading to inhibition in the proliferation of CRC cells. These data support the ability of NAC to work as a chemo-preventive agent [40]. Based on these results, we are hypothesizing that the significant elevation in NAC levels in our vehicle controls (19.134-fold) compared to the negative ones was a preventive method against tumor growth and angiogenesis.

The steroid hormone cortisol, commonly known as the ‘body stress hormone’ [46], was significantly increased in the vehicle controls (18.404-fold) compared to negative controls. High cortisol levels have been associated with worse prognosis in patients with various types of malignancies[16]. The sympathetic nervous system (SNS) and the hypothalamic-pituitary-adrenal (HPA) axis are stimulated by psychological processes, increasing the secretion of stress-related neurohormonal mediators like cortisol and catecholamines. Also, cortisol has been demonstrated to contribute to tumorigenesis and the proliferation of cancer cells by interfering with DNA repair and promoting DNA damage[35]. In addition, it has been found that psychological stress in xenograft cancer models can increase systemic and tumor levels of cortisol, and also significantly promote xenograft tumor growth [48]. These data prove that cortisol is a potential biomarker for cancers and xenograft cancer models specifically.

The impact of trans-fatty acids (TFAs) on cancers has lately been recognized. A previous study found that elaidic acid (EA), a trans-fatty acid, improves CRC cells' growth, survival, and metastasis. Ohmori and colleagues examined the effect of EA on two CRC cell lines, CT26 and HT29. CRC cells were treated with EA, and other same cells were treated with vehicle. They demonstrated that EA-treated CRC cells dramatically increased tumor development and metastasis in the lung, liver, and peritoneum compared to CRC cells treated with vehicle. Also, EA-treated cells produced more evident amounts of CRC cell spheres than vehicle-treated cells did [41]. Their findings support our results that show a significant increase in the amount of EA in the vehicle controls (10.677-fold) compared to negative controls, which show vital implications for the diagnosis of CRC.

Ethanolamine, also known as monoethanolamine (MEA) was significantly elevated in the vehicle controls compared to negative controls (1.6767-fold). It was revealed in previous work that MEA may improve colonic barrier function in inflammatory bowel disease (IBD) patients by increasing antimicrobial protein secretion. Also, it was found that MEA can alleviate colonic inflammation by increasing the secretion of secretory IgA (SIgA) [60], which plays an important role in the protection and homeostatic regulation of intestinal, respiratory, and urogenital mucosa, and works as the first line of defense in defending the intestinal epithelium from pathogenic microorganisms and enteric toxins[21]. These results support our hypothesis that the elevated concentrations of MEA were providing an anti-inflammatory effect to our vehicle controls.

### 4.2 Steroidogenesis

The steroidogenesis process was altered when comparing vehicle controls with negative controls (Fig. 9A). Steroidogenesis is the process which converts cholesterol into physiologically active steroid hormones[10]. Preclinical findings demonstrate that endogenous sex steroid hormones may play a role in the development of CRC. High serum cholesterol levels have been associated with a greater chance of developing malignancies such as colon, rectal, testicular and prostate cancer [13]. A previous study revealed that cholesterol promoted colon cancer growth in mice models [14]. Another research found that cholesterol stimulated cell proliferation and inhibited apoptosis in CRC, and both the colony forming abilities and cell viability were improved in HCT-116 cells following cholesterol treatment [55].

Some in vitro studies have suggested that estradiol could have tumorigenic effects on colorectal cells[8]. Male mice studies also suggest that testosterone, through its tumor-promoting actions, may play a role in the development of colonic adenomas. A possible indirect pathway for this action is an increase in the stress hormones cortisol, which affects the tumor environment [5]. In our study, the steroidogenesis process was mainly impacted by aldosterone, cortisol, and tetrahydrocortisone, a down-stream metabolite of cortisol and cortisone, which were all elevated in our vehicle models (32.131-fold, 18.404-fold, and 8.1298-fold respectively). We hypothesize that cholesterol level was elevated in our vehicle models, which led to the elevation of these three steroid hormones, affecting tumor growth.

### 4.3 Phosphatidylethanolamine Biosynthesis

Phosphatidylethanolamine Biosynthesis was significantly enriched when vehicle controls and negative controls were compared (Fig. 9A). Phosphatidylethanolamine (PE) is a common glycerophospholipid in eukaryotic cells. It is a major lipid component of cellular membranes in a variety of organisms, which is necessary for mammalian development[9]. CRC cells that are highly proliferative have a high demand and necessity for new building blocks, of which a significant portion is used to build cellular membranes. Cellular membranes' altered structure and function reveal phospholipid metabolism dysregulation in tumors. These alterations have been linked to tumor growth and poor patient outcomes in a number of cancers, including CRC [51]. Also, previous studies revealed that alterations in phospholipids correlate with CRC development [42], [45]. In our study, phosphatidylethanolamine biosynthesis was mainly influenced by ethanolamine, which was elevated in vehicle control group.

### 4.4 Potential biomarkers for CRC treatment

Nowadays, metabolomic research is not only employed in the identification of diagnostic cancer biomarker, but also to monitor metabolic response during therapeutic intervention [7]. T-test carried out between potential responders’ group (4 models) and vehicle controls (5 models) revealed that there are 20 significantly altered metabolites between them. 17 metabolites were elevated and 3 were reduced in the potential responders group compared to vehicle controls.

Notably, pyridoxine, also known as vitamin B6, has been greatly associated with CRC. It is engaged in over 100 coenzyme reactions and may reduce oxidative stress, inflammation, and cell proliferation as well as the risk of CRC (X.-H. [58]). Our results showed that pyridoxine was significantly elevated in potential responders (5.3084-fold) compared to the vehicle controls, emphasizing the probability of its association with reducing CRC growth. Furthermore, vitamin B6 has been proven to reduce angiogenesis and lower nitric oxide, both of which relate to carcinogenesis inhibiting. Furthermore, it was reported that low vitamin B6 levels may be linked to chronic inflammation, which is a risk factor for CRC[32]. Our finding agrees with another study that also revealed that supplemental vitamin B6 was effective in suppressing cell proliferation and reducing the number of colon tumors in animal models(X.-H. [58]).

A naturally occurring amino acid, chemical precursor, and metabolic intermediary in the manufacture of the neurotransmitter serotonin is 5-hydroxytryptophan (L-5-HTP). It is produced in the body from tryptophan amino acid. 5-hydroxytryptophan level was significantly higher in the potential responders’ group compared to vehicle controls (2.5367-fold). A previous study investigated using animal models for any potential in vivo anticancer effects of L-5-HTP. Mouse colon cancer cell lines were subcutaneously implanted. It was found that L-5-HTP therapy significantly slowed the growth of colon tumors and decreased tumor mass when compared to vehicle control. Also, L-5-HTP showed significantly better survival. These effects were a result of inhibiting PD-L1 expression, which binds to PD-1 receptor. PD-L1 is overexpressed in the tumor microenvironment to help escape immune attack. The PD-1/PD-L1 signaling axis can be inhibited as a possible method to restore the immunosuppressive tumor microenvironment (J. [24]). These findings support our results, indicating the likelihood of L-5-HTP being a potential biomarker for CRC treatment.

Serotonin (5-HT) is a powerful neuronal, peripheral, and gastric signaling chemical [38]. Interestingly, it was revealed that 5-HT has an interesting dual effect in CRC. There is significant evidence that 5-HT activity could preserve the colon from early CRC-related events. It was revealed in a previous study that 5-HT was able to reduce blood flow in the initiation steps of CRC by constricting arterioles[28]. Third study found that the blockage of 5-HT reuptake by fluoxetine not only elevated 5-HT levels in the colon, but also gave a preventive effect against colon cancer by reducing the development of lesions inside the colon's preneoplastic milieu in the initiation steps, using rats model [29]. Moreover, another study used mice models and found a novel protective effect of serotonin by promoting DNA repair in the early stages of colorectal carcinogenesis [47]. On the other hand, 5-HT has been thought to promote CRC proliferation for a few decades. Xia et al. found that a high level of 5-HT in advanced-stage CRC patients was associated with advanced tumor metastasis, leading to poor overall survival [56]. Arisha and colleagues discovered that while fenitrothion therapy increased free radical levels, DNA damage, and apoptosis, there was a significant drop in 5-HT levels [3]. Additionally, It was found in a previous study that 5-HT deficiency led to slower cancer cells growth [30]. These findings align with our results, leading us to hypothesize that 5-HT, which may promote CRC progression, was diminished following treatment with topotecan.

### 4.5 Tryptophan metabolism

One of the essential amino acids for humans is tryptophan (TRP). It has a variety of nutritional and physiological purposes, making it a crucial functional amino acid. In animals, intestinal immune homeostasis is significantly influenced by endogenous TRP metabolites. According to the literature, several disorders, including gastrointestinal diseases, are associated with TRP metabolism disorders. Numerous investigations conducted recently have revealed that TRP metabolism and tumor growth are tightly connected [6]. In-vivo, serotonin metabolism and kynurenine metabolism are the two major metabolic pathways in which TRP is involved. 90 % of TRP is metabolized into the kynurenine (KYNA) metabolic pathway (H. L. [59]). It was found in a previous study that kynurenine in kynurenine metabolic pathway is significantly elevated in colon cancer cells and promotes cells proliferation. In addition, it was reported that TRP metabolites reduce inflammation *in vivo* and restore the intestinal wall structure, potentially delaying the progression of CRC (S. [23]). In our study, TRP metabolism was mainly impacted by two TRP metabolites, serotonin and 5-hydroxytryptophan.

### 4.6 Potential biomarkers for treatment response prediction

When T-test analysis was performed between both treated xenograft groups, potential responders and potential non-responders, six metabolites were identified as statistically significant. Four metabolites were elevated in the potential responder compared to potential non-responders, including 7-ketocholesterol, 2-oxoarginine, pyridoxine, and L-proline. In addition, serotonin and succinylacetone were identified as statistically reduced metabolites in the potential responder xenografts compared to potential non-responders. These metabolites can be utilized as predictive biomarkers, which are biological features that predict the degree of improvement a patient can receive from a treatment compared to baseline condition [49].

### 4.7 Potential biomarkers for safety

Student’s *t*-test analysis between positive and negative controls revealed only one significant reduced metabolite in the positive controls, compared to negative ones, which is homovanillic acid (HA) (0.42-fold). Although topotecan is widely used, we can employ this metabolite to indicate the safety of topotecan in our setting. As previously mentioned, HA is a major terminal metabolite of dopamine, so low HA levels reflect low dopamine levels. Symptoms related with this condition are sleep disturbances, depression, and fatigue. Notably, once vehicle controls and negative controls were compared, HA was also reduced significantly. So, further studies are needed to confirm the correlation between HA and CRC.

## 5 Conclusions, limitations, and future work

Our study highlights topotecan-induced changes in several metabolites and metabolic pathways in HCT-116 xenograft model, which could significantly contribute to translational cancer research. These changes may facilitate the identification of novel diagnostic biomarkers for noninvasive cancer diagnosis and subsequent secondary cancer prevention. Moreover, we identified novel predictive biomarkers that may estimate the improvement a patient could experience from treatment relative to baseline conditions.

However, the study presents certain limitations. A relatively small number of animals were used per group, and CRC cells were injected subcutaneously rather than directly into the colon or rectum. Due to the small animal sample, it was not possible to collect blood samples at multiple time points for early biomarker detection. Additionally, only one standard cell line of CRC was used. Expanding the number of animal models is also recommended to more effectively validate the implications of the identified metabolites and metabolic pathways on CRC, as well as their diagnostic and predictive value. Furthermore, as this study serves as a preliminary investigation, our future studies aim to validate key metabolites through additional experimental approaches to confirm their biological significance and potential role as therapeutic or diagnostic markers in CRC.

## CRediT authorship contribution statement

**El-Huneidi Waseem:** Writing – review & editing. **Ramadan Wafaa:** Writing – review & editing. **Abuhelwa Ahmad:** Writing – review & editing. **El-Awady Raafat:** Writing – review & editing. **Hamoudi Rifat:** Writing – review & editing. **Qaisar Rizwan:** Methodology. **Khan Farman:** Methodology. **Semreen Mohammad:** Writing – review & editing, Methodology, Conceptualization. **Al-Hroub Hamza:** Writing – review & editing. **Bustanji Yasser:** Writing – review & editing. **Soares Nelson:** Writing – review & editing, Methodology, Conceptualization. **Alzoubi Karem:** Writing – review & editing. **Zenati Ruba A.:** Writing – review & editing, Writing – original draft, Methodology. **Abu-Gharbieh Eman:** Writing – review & editing. **Hagyousif Yousra:** Writing – review & editing, Writing – original draft, Methodology.

## Funding

The authors would like to acknowledge The University of Sharjah financial support. This study was financially supported by the University of Sharjah (competitive grant number 2201110155 and targeted grant number 24011101105).

## Declaration of Competing Interest

The authors declare that they have no known competing financial interests or personal relationships that could have appeared to influence the work reported in this paper.

## Data Availability

Metabolomics Workbench repository (DOI: http://dx.doi.org/10.21228/M8VB2V) with data ID ST003201.
