# Supplementary material for: Molecular signatures of xenograft colorectal cancer in mice treated with topotecan: A mass spectrometry-based study
Source: Toxicol Rep. 2025 May 14;14:102045. doi: 10.1016/j.toxrep.2025.102045 (PMC12149579; doi:10.1016/j.toxrep.2025.102045)
Supplement: Supplementary file 1 — Supplementary material [file mmc1.docx]

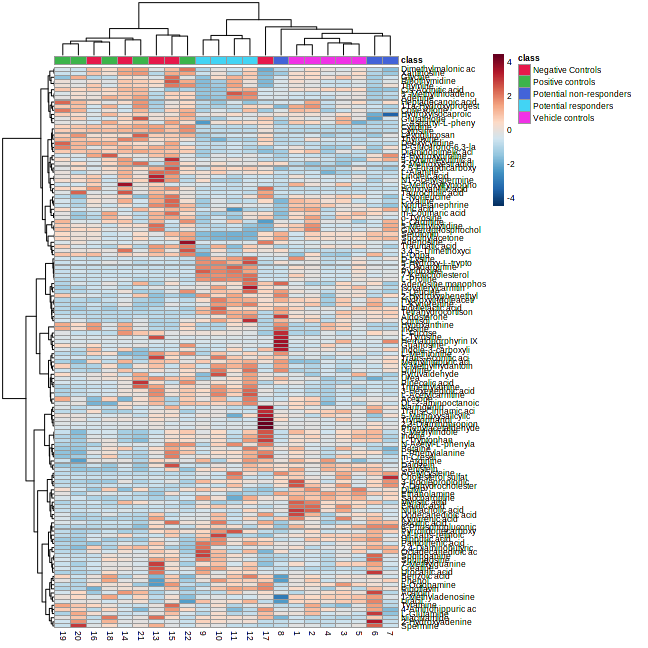
**Figure S1.** Heat map representing the identified metabolites in the 5 groups; potential responders, potential non-responders, vehicle controls, positive controls and negative controls.

| Weights for HCT-116 Xenograft Models and Positive Controls | | | | | | | | | | | | | | | | | | | | |
| --- | --- | --- | --- | --- | --- | --- | --- | --- | --- | --- | --- | --- | --- | --- | --- | --- | --- | --- | --- | --- |
|  | Day 1 (HCT-116 cells injection) | Day of first injection of topotecan |  | | | | | | | | | | | Day of dissection | | Tumor weight | | Animal weight without tumor | Total weight lost during the study | |
|  | 12-Dec | 9-Jan | 11-Jan | 13-Jan | 16-Jan | 18-Jan | 20-Jan | 23-Jan | 25-Jan | 27-Jan | 30-Jan | 1-Feb | 3-Feb | 6-Feb |  | | | | | |
| Untreated xenografts (vehicle controls) | | | | | | | | | | | | | | | | | | | | |
| Animal 1 | 30.2 g |  | Average weight: 30.1 g | | | | | | | | | | | | | 9.5 g | | 21.4 g | 8.8 g | |
| Animal 2 | 30.7 g |  | Average weight: 32.3 g | | | | | | | | | | | | | 11.2 g | | 19.5 g | 11.2 g | |
| Animal 3 | 30.5 g |  | Average weight: 31.2 g | | | | | | | | | | | | | 7.2 g | | 24.4 g | 6.1 g | |
| Animal 4 | 25.6 g |  | Average weight: 27 g | | | | | | | | | | | | | 10.3 g | | 20.7 g | 4.9 g | |
| Animal 5 | 26.7 g |  | Average weight: 29 g | | | | | | | | | | | | | 12.7 g | | 18.2 g | 8.5 g | |
| Treated xenografts (topotecan models) | | | | | | | | | | | | | | | | | | | | |
| Animal 1 | Average weight: 26.7 g | | | | | | | | | | | | | | | 8.3 g | | 23.5 g | 3.2 g | |
| Animal 2 | Average weight: 28.7 g | | | | | | | | | | | | | | | 3.2 g | | 25.8 g | 2.5 g | |
| Animal 3 | Average weight: 27.3 g | | | | | | | | | | | | | | | 4.8 g | | 20.5 g | 6.8 g | |
| Animal 4 | Average weight: 28.7 g | | | | | | | | | | | | | | | 5.4 g | | 26.0 g | 2.5 g | |
| Animal 5 | Average weight: 28.9 g | | | | | | | | | | | | | | | 5.7 g | | 25.6 g | 5.0 g | |
| Animal 6 | Average weight: 29.5 g | | | | | | | | | | | | | | | 7.8 g | | 23.4 g | 6.1 g | |
| Animal 7 | Average weight: 28.1 g | | | | | | | | | | | | | | | 7.6 g | | 20.2 g | 7.7 g | |
| Positive controls (injected with topotecan) | | | | | | | | | | | | | | | | | | | | |
| Animal 1 | Average weight: 26.1 g | | | | | | | | | | | | | | | |  | | | 1.9 g |
| Animal 2 | Average weight: 28.8 g | | | | | | | | | | | | | | | |  |  |  | 1.0 g |
| Animal 3 | Average weight: 27.0 g | | | | | | | | | | | | | | | |  |  |  | 1.1 g |
| Animal 4 | Average weight: 27.4 g | | | | | | | | | | | | | | | |  |  |  | 0.8 g |
| Animal 5 | Average weight: 26.9 g | | | | | | | | | | | | | | | |  |  |  | 1.0 g |

**Table S1.** Weights for HCT-116 xenograft models and positive controls throughout the study.
