# Supplementary material for: Molecular signatures of xenograft colorectal cancer in mice treated with topotecan: A mass spectrometry-based study
Source: Toxicol Rep. 2025 May 14;14:102045. doi: 10.1016/j.toxrep.2025.102045 (PMC12149579; doi:10.1016/j.toxrep.2025.102045)
Supplement: Supplementary file 2 — Supplementary material [file mmc2.pdf]

NOTE: Please save this file locally before filling in the table, DO NOT work on the file within your internet browser as changes will not be saved. Adobe Acrobat Reader (available free here) is recommended for completion.

The ARRIVE guidelines 2.0: author checklist

The ARRIVE Essential 10

These items are the basic minimum to include in a manuscript. Without this information, readers and reviewers cannot assess the reliability of the findings.

Item		Recommendation	Section/line number, or reason for not reporting
**Study design**	1	For each experiment, provide brief details of study design including:	
		a. The groups being compared, including control groups. If no control group has been used, the rationale should be stated.	**a.** The study compared four distinct groups of mice: CRC xenograft mice injected with topotecan, CRC xenograft mice injected with vehicle control, healthy mice as a negative control, and healthy mice injected with topotecan as a positive control. These groups are described in detail in the manuscript in the Materials and Methods **Section 2.2 on line 127.**
		b. The experimental unit (e.g. a single animal, litter, or cage of animals).	**b**. The experimental unit, identified as the individual mouse as described in detail in the manuscript in the Materials and Methods **Section 2.2, line 127-144**.
**Sample size**	2	a. Specify the exact number of experimental units allocated to each group, and the total number in each experiment. Also indicate the total number of animals used.	**a**. The exact number of experimental units allocated to each group are as follows: CRC xenograft mice injected with topotecan (n = 7), CRC xenograft mice injected with vehicle control (n = 5), healthy mice as a negative control (n = 5), and healthy mice injected with topotecan as the positive control (n = 5). In total, 22 animals were used in the experiment, as described in Materials and Methods **Section 2.2, line 127.**
		b. Explain how the sample size was decided. Provide details of any *a priori* sample size calculation, if done.	**b.** The sample size was not explicitly calculated using a priori methods, but the study employed a relatively small number of animals per group. This limitation is acknowledged in the manuscript's **Section 5, line 637.**
**Inclusion and exclusion criteria**	3	a. Describe any criteria used for including and excluding animals (or experimental units) during the experiment, and data points during the analysis. Specify if these criteria were established *a priori.* If no criteria were set, state this explicitly.	**a.** In the manuscript, no specific criteria for including or excluding animals or experimental units during the experiment were explicitly established a priori. All 22 animals used in the study were included in the experimental design, and no exclusion criteria were mentioned in **Section 2.2, line 127-144.**
		b. For each experimental group, report any animals, experimental units or data points not included in the analysis and explain why. If there were no exclusions, state so	**b.** All animals were included in the analysis, and no animals or data points were excluded from any experimental group mention in **Section 2.5, line 168.**
		c. For each analysis, report the exact value of *n* in each experimental group.	**c.** The number of experimental animals in each group and their allocation had been described in **Section 2.2, line 127.**
**Randomisation**	4	a. State whether randomisation was used to allocate experimental units to control and treatment groups. If done, provide the method used to generate the randomisation sequence.	**a**. The experimental animals were numbered, and randomly grouped by number as described in **Section 2.2, line 127-144.**
		b. Describe the strategy used to minimise potential confounders such as the order of treatments and measurements, or animal/cage location. If confounders were not controlled, state this explicitly.	**b.** To reduce potential disturbance, laboratory animals were housed in standard animal rooms, and fed to SPF grade**.** as described in **Section 2.2, line 140-144.**
**Blinding**	5	Describe who was aware of the group allocation at the different stages of the experiment (during the allocation, the conduct of the experiment, the outcome assessment, and the data analysis).	Under the guidance of corresponding author, the first six authors assisted to complete the experiment, including experiment allocation, experiment implementation, experiment result evaluation and data analysis, while the remaining authors assisted in writing and reviewing the article.

**Outcome measures**	6	a. Clearly define all outcome measures assessed (e.g. cell death, molecular markers, or behavioural changes).	**a.** The outcome measures in the study include changes in tumor weight, animal weight, and metabolic profiles assessed through UHPLC-ESI-QTOF-MS. Tumor growth was monitored by measuring tumor masses in xenograft models, with statistical significance being determined through independent t-tests. Metabolic alterations were evaluated, including significant changes in 53 metabolites across the four groups, with specific attention to biomarkers indicative of response to topotecan treatment. As described in **Section 3.1, line 274-291, 3.2, line 310-319, and 3.3 line 332–357.**
		b. For hypothesis-testing studies, specify the primary outcome measure, i.e. the outcome measure that was used to determine the sample size.	**b.** The primary outcome measure for hypothesis testing in this study was the tumor weight difference between treated xenografts and vehicle controls. This measure was used to evaluate the efficacy of topotecan in reducing tumor growth, as indicated by the significant reduction in tumor weight in the treated group compared to controls ($p = 0.0049$) as described in **Section 3.1, line 274-291.**
**Statistical methods**	7	a. Provide details of the statistical methods used for each analysis, including software used.	**a.** The statistical analysis in the study was performed using, one-way ANOVA for comparisons among the four groups, and independent two-tailed t-tests were used for pairwise comparisons between the treated and control groups. The significance level was set at a q-value of <0.05, and fold changes were adjusted at 1.5. The false discovery rate (FDR) method was applied to correct for multiple hypothesis testing and to reduce the likelihood of false positives as described **in Section 2.9, line 244-271.**
		b. Describe any methods used to assess whether the data met the assumptions of the statistical approach, and what was done if the assumptions were not met.	**b.** The assumptions of normality and homogeneity of variances were assessed visually using histograms and boxplots. Histograms were used to check whether the data within each group followed a normal distribution, while boxplots were employed to evaluate the spread of the data and detect any violations of the equal variance assumption **Section 2.9, line 244-271.**
**Experimental animals**	8	a. Provide species-appropriate details of the animals used, including species, strain and substrain, sex, age or developmental stage, and, if relevant, weight.	**a.** All animals used in the study were 10-week-old male BALB/c nude mice, weighing between 26 g and 28 g. These mice were housed in standard laboratory conditions, and their behavior and health were monitored daily. Their weights were recorded every other day during the study**.** This study was approved by the Institutional Animal Care and Use Committee (IACUC) at the University of Sharjah (Ethical approval number: ACUC-06-08-2022). As described in **Section 2.2, line 127-144.**
		b. Provide further relevant information on the provenance of animals, health/immune status, genetic modification status, genotype, and any previous procedures.	**b.** The BALB/c nude mice were maintained under SPF (Specific Pathogen-Free) grade conditions to ensure their health and immune status. The mice were obtained from a specific institutional animal facility and had no prior genetic modifications or procedures. All mice were housed in standard animal rooms to reduce potential disturbances, and all procedures followed ethical guidelines under the approval of the Institutional Animal Care and Use Committee (IACUC). No previous procedures were conducted on these animals prior to the experiment. As described in **Section 2.2, line 127-144.**
**Experimental procedures**	9	For each experimental group, including controls, describe the procedures in enough detail to allow others to replicate them, including:<br>a. What was done, how it was done and what was used.<br>b. When and how often.<br>c. Where (including detail of any acclimatisation periods).<br>d. Why (provide rationale for procedures).	The experimental process, methods and results had been described in **Section Materials and methods, line 117.** Anyone can repeat this research at any time and place.<br>**a. Section 2.2, 2.3, and 2.4**<br>**b. Section 2.3**<br>**c. Section 2.2**<br>**d. Section 2.5, 2.6. 2.7, 2.8 and 2.9**

**Results**	10	For each experiment conducted, including independent replications, report:<br>a. Summary/descriptive statistics for each experimental group, with a measure of variability where applicable (e.g. mean and SD, or median and range).	**a.** The statistical analysis mentioned focuses on p-values and general comparisons without including detailed summary statistics such as the standard deviation (SD) for the tumor weight.
		b. If applicable, the effect size with a confidence interval.	**b.** No applicable. Our study strictly followed the ARRIVE guidelines and minimized the impact on experimental results.

The Recommended Set

These items complement the Essential 10 and add important context to the study. Reporting the items in both sets represents best practice.

Item		Recommendation	Section/line number, or reason for not reporting
**Abstract**	11	Provide an accurate summary of the research objectives, animal species, strain and sex, key methods, principal findings, and study conclusions.	We had described the research objectives, main methods and findings, and conclusions in **Section Abstract, line 41-61.**
**Background**	12	a. Include sufficient scientific background to understand the rationale and context for the study and explain the experimental approach.	**a.** The background of this research had been explained in **Section Introduction, line 67-116, Discussion, line 421-629 and Conclusions, line 631-643.**
		b. Explain how the animal species and model used address the scientific objectives and, where appropriate, the relevance to human biology.	**b.** The animal model used in the study was male BALB/c nude mice, aged 10 weeks, which are immunocompromised. This species and strain were chosen because they are commonly used in cancer research, especially in xenograft models where human cancer cells are implanted. The use of the HCT-116 human colorectal carcinoma cell line in this model allows for the replication of CRC tumor growth in a controlled environment. The relevance to human biology is significant because the HCT-116 cells are representative of human CRC, and the BALB/c nude mice lack an immune response, making them ideal for studying human tumor growth without rejection.
**Objectives**	13	Clearly describe the research question, research objectives and, where appropriate, specific hypotheses being tested.	Research questions and objectives were clearly described in **Section Introduction, line 67-116.**
**Ethical statement**	14	Provide the name of the ethical review committee or equivalent that has approved the use of animals in this study, and any relevant licence or protocol numbers (if applicable). If ethical approval was not sought or granted, provide a justification.	The study was approved by the Institutional Animal Care and Use Committee (IACUC) at the University of Sharjah. The ethical approval number for this study is ACUC-06-08-2022. The Ethical statement was described in **Section 2.2, line 127-144.**
**Housing and husbandry**	15	Provide details of housing and husbandry conditions, including any environmental enrichment.	The experimental animals were housed in standard animal rooms to minimize potential disturbances. The mice were maintained under Specific Pathogen-Free (SPF) conditions to ensure their health throughout the study. Daily welfare assessments were conducted, focusing on cage environment, animal behavior, and physical appearance. Food and drink were accessible from the cage floor to encourage eating after each injection. The animals were acclimatized to these conditions prior to the start of the experimental procedures as described in **Section 2.2, line 127-144.**

**Animal care and monitoring**	16	a. Describe any interventions or steps taken in the experimental protocols to reduce pain, suffering and distress.	**a.** To reduce pain, suffering, and distress in the experimental mice, daily welfare assessments were conducted, focusing on the animals' cage environment, behavior, and physical appearance. Additionally, the injections of topotecan and vehicle control were administered intraperitoneally with care to minimize discomfort, and food and drink were made easily accessible from the cage floor to encourage normal eating behavior after injections as described in **Section 2.2, line 127-144.**
		b. Report any expected or unexpected adverse events.	**b.** The manuscript does not report any unexpected adverse events during the study. It notes that positive control animals experienced negligible weight loss, suggesting that the dose of topotecan used was safe and well-tolerated. Our study strictly followed the ARRIVE guidelines, and fully concerned about the safety of laboratory animals.
		c. Describe the humane endpoints established for the study, the signs that were monitored and the frequency of monitoring. If the study did not have humane endpoints, state this.	**c.** In this study, humane endpoints were established to minimize animal suffering. The animals were monitored daily for signs of distress, including severe weight loss, labored breathing, abnormal behavior, and lethargy. If any of these signs were observed, the animal was humanely euthanized in accordance with institutional guidelines.
**Interpretation/ scientific implications**	17	a. Interpret the results, taking into account the study objectives and hypotheses, current theory and other relevant studies in the literature.	**a**. In the section discussion and Conclusions/line 1-5, the results were fully explained in combination with literature reports
		b. Comment on the study limitations including potential sources of bias, limitations of the animal model, and imprecision associated with the results.	**b**. The study faced limitations, including the small number of animals per group and the subcutaneous rather than orthotopic injection of CRC cells. The small sample size also prevented the collection of blood samples at multiple time points for early biomarker detection. Furthermore, only one standard CRC cell line was used. Expanding the number of animal models is recommended to better validate the identified metabolites and metabolic pathways, as well as their diagnostic and predictive value in CRC as described in **Conclusion, line 631-643.**
**Generalisability/ translation**	18	Comment on whether, and how, the findings of this study are likely to generalise to other species or experimental conditions, including any relevance to human biology (where appropriate).	This study is a basic study, and preliminary results. Whether the results can be applied to other species or experimental conditions requires further validation.
**Protocol registration**	19	Provide a statement indicating whether a protocol (including the research question, key design features, and analysis plan) was prepared before the study, and if and where this protocol was registered.	A protocol was prepared before the study without registration.
**Data access**	20	Provide a statement describing if and where study data are available.	Metabolomics data are deposited in Metabolomics Workbench with study ID ST003201 (DOI: http://dx.doi.org/10.21228/M8VB2V).
**Declaration of interests**	21	a. Declare any potential conflicts of interest, including financial and non-financial. If none exist, this should be stated.	**a.** The authors have no competing interests to declare (described in **Section Disclosure Statement, line 674**).
		b. List all funding sources (including grant identifier) and the role of the funder(s) in the design, analysis and reporting of the study.	**b**. This study was supported by 2 fundings. This study was financially supported by the University of Sharjah (competitive grant number 2201110155 and targeted grant number 2301101775) described in **Section Funding, line 681-683**).
